# Supplementary material for: Tumorigenicity decrease in Bcl-xL deficient MDCK cells ensuring the safety for influenza vaccine production
Source: PLoS One. 2024 Dec 16;19(12):e0311069. doi: 10.1371/journal.pone.0311069 (PMC11649150; doi:10.1371/journal.pone.0311069)
Supplement: S1 File — (ZIP) [file pone.0311069.s002.zip › S2_File/4_KOG_enrichment/barplot/B_vs_A.up_KOG_categorie.pdf]

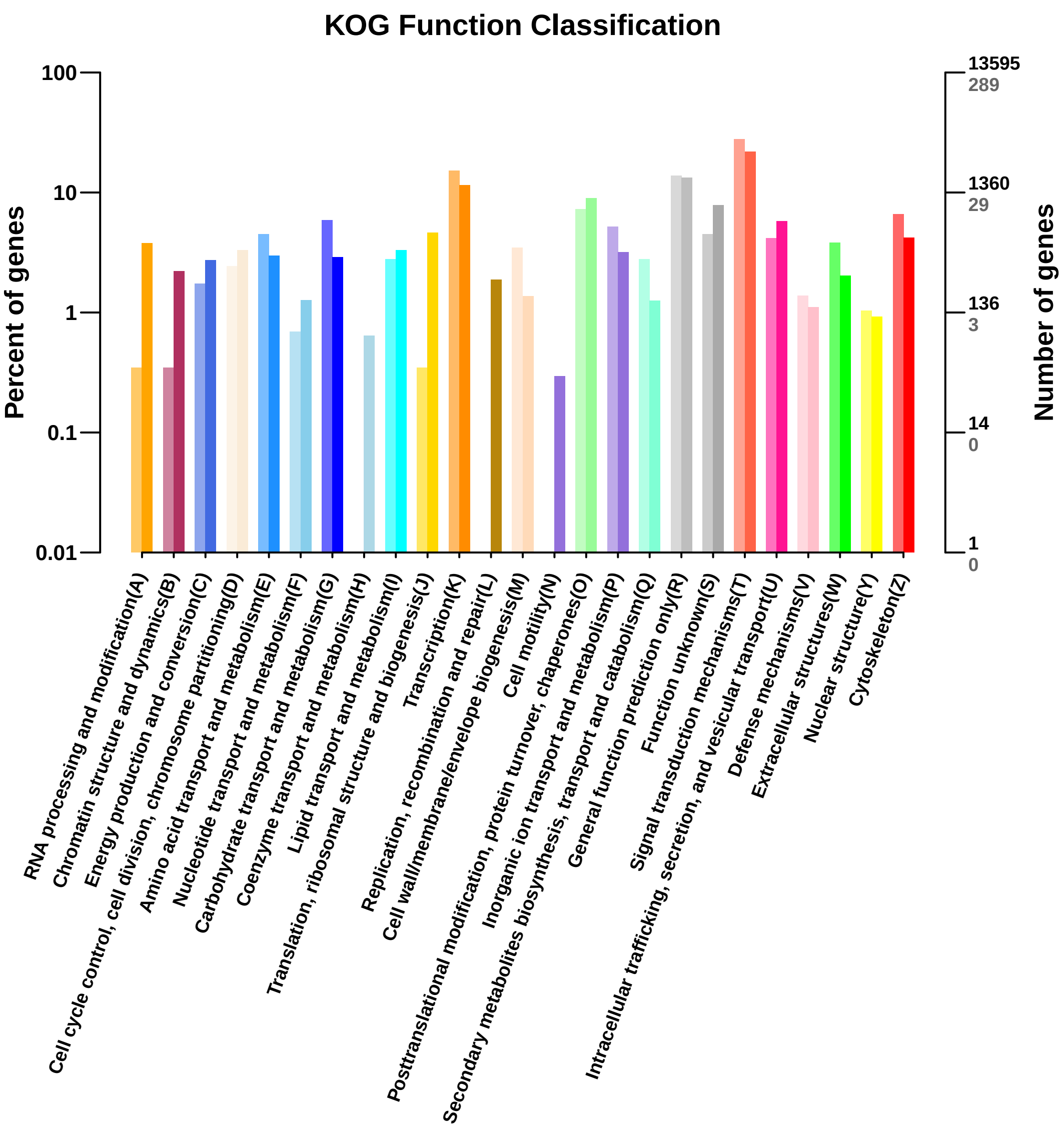

KOG Function Classification
Percent of genes
Number of genes
100
10
1
0.1
0.01
13595
289
1360
29
136
3
14
0
1
0
RNA processing and modification(A)
Chromatin structure and dynamics(B)
Energy production and conversion(C)
Cell cycle control, cell division, chromosome partitioning(D)
Amino acid transport and metabolism(E)
Nucleotide transport and metabolism(F)
Carbohydrate transport and metabolism(G)
Coenzyme transport and metabolism(H)
Lipid transport and metabolism(I)
Translation, ribosomal structure and biogenesis(J)
Transcription(K)
Replication, recombination and repair(L)
Cell wall/membrane/envelope biogenesis(M)
Cell motility(N)
Posttranslational modification, protein turnover, chaperones(O)
Inorganic ion transport and metabolism(P)
Secondary metabolites biosynthesis, transport and catabolism(Q)
General function prediction only(R)
Function unknown(S)
Signal transduction mechanisms(T)
Intracellular trafficking, secretion, and vesicular transport(U)
Defense mechanisms(V)
Extracellular structures(W)
Nuclear structure(Y)
Cytoskeleton(Z)
